# Unilateral Presentation of Vogt-Koyanagi-Harada Syndrome

**DOI:** 10.18502/jovr.v15i1.5954

**Published:** 2020-02-02

**Authors:** Seyedeh Maryam Hosseini, Maryam Dourandish, Marjan Mazouchi

**Affiliations:** ^1^Eye Research Center, Mashhad University of Medical Sciences, Mashhad, Iran; ^2^Eye Research Center, Matini Eye Hospital, Kashan University of Medical Sciences, Kashan, Iran

**Keywords:** Asymmetric VKH, Enhanced-Depth Optical Coherence Tomography, Indocyanine Green Angiography, Vogt-Koyanagi-Harada Disease

## Abstract

**Purpose:**

To report a case of acute Vogt-Koyanagi-Harada (VKH) disease with unilateral clinical manifestations followed by late fellow eye involvement.

**Case Report:**

This case report reviews the 12-month follow-up observation of a 44-year old woman who presented to the emergency department with unilateral progressive and painless visual blurring. Ophthalmoscopic findings, best-corrected visual acuity (BCVA), fluorescein angiography (FAG), enhanced-depth optical coherence tomography (EDI-OCT), indocyanine green angiography, and response to treatment were evaluated. Her BCVA was 20/50 (logMAR: 0.4) in the right eye and 20/20 (logMAR: 0) in the left eye. Eye examination revealed optic disc swelling and multiple serous retinal detachments in the right eye and a normal left eye. She had headache, dysacusia, and mild hearing problem. Her past ocular and drug histories were unremarkable. Retinal imaging revealed characteristic features of VKH in the right eye. All laboratory testing results were inconclusive. VA and OCT findings significantly improved following the treatment with methylprednisolone 1 g/day continued by tapering dose of oral prednisolone. Two months after the presentation and during prednisolone tapering, VA of the left eye decreased and fundus examination revealed multiple serous retinal detachments in this eye.

**Conclusion:**

Ophthalmologists should recognize unilateral and asymmetrical VKH disease with subtle systemic involvement.

##  INTRODUCTION

Vogt-Koyanagi-Harada disease (VKH) is a rare, chronic, inflammatory granulomatous multisystem disorder of unknown cause that is characterized by bilateral panuveitis, iridocyclitis, exudative retinal detachment, and optic disc swelling. The disorder affects melanocyte-rich tissues, including the eyes, auditory system, meninges, skin, and hair.^[1,2,3,4,5]^

The diagnosis of VKH is based on the Revised Diagnostic Criteria (RDC) of 2001.^[6,7]^ The disease is classified into three categories: complete, incomplete, and probable VKH.^[6]^

The disease is divided into four consecutive stages, namely prodromic, acute uveitic, convalescent, and chronic.^[7]^ Approximately 17–73% of cases progress to chronic stage.^[8]^ It usually affects both eyes simultaneously.^[1,6]^ However, unilateral ocular involvement at onset, followed by later involvement of the second eye in 2–3 weeks is reported in 30% of the cases.^[7,9]^ Reports of long-term follow-up in patients with unilateral ocular disease are few.^[8,9,10,11]^

Ocular imaging modalities, including fluorescein (FA) and indocyanine angiography (ICGA); optical coherence tomography (OCT), especially enhanced-depth imaging OCT (EDI-OCT); and ultrasonographic findings are useful in the diagnosis and follow-up of patients.^[6]^

Many reports have indicated that patients may not manifest all diagnostic criteria but seem to have the disease.^[1]^ Herein, we report an interesting case of clinically unilateral probable VKH with subclinical fellow eye involvement on ICGA, which progressed to clinical involvement later.

##  CASE PRESENTATION

**Figure 1:**
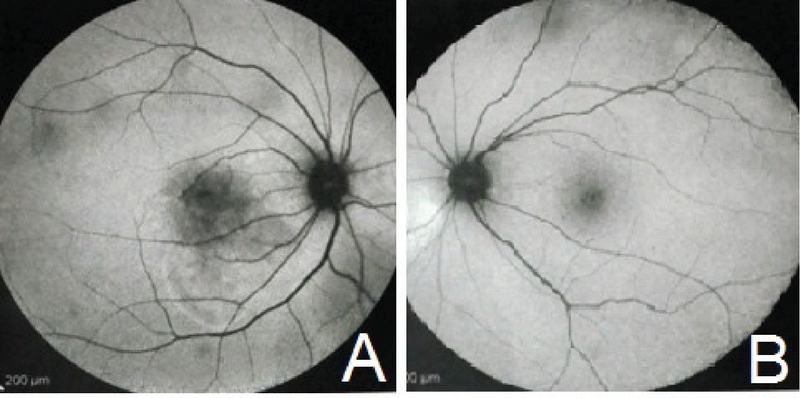
Fundus auto-fluorescence (FAF). (A) Hypo-autofluorescence area in macular area and hyper-auto-fluorescence ring in inferior of macula in the right eye. (B) Normal FAF in the left eye.

**Figure 2:**
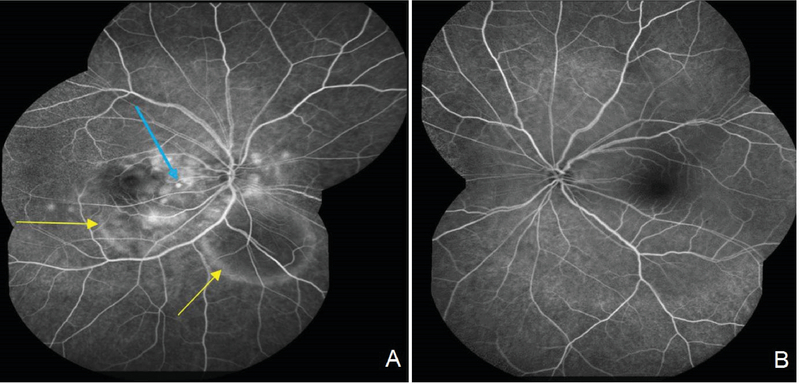
Fluorescein angiography at presentation (arteriovenous phase). (A) Multifocal areas of pinpoint leakage (blue arrow), pooling (yellow arrows) staining (right eye ). (B) Normal in the left eye.

**Figure 3:**
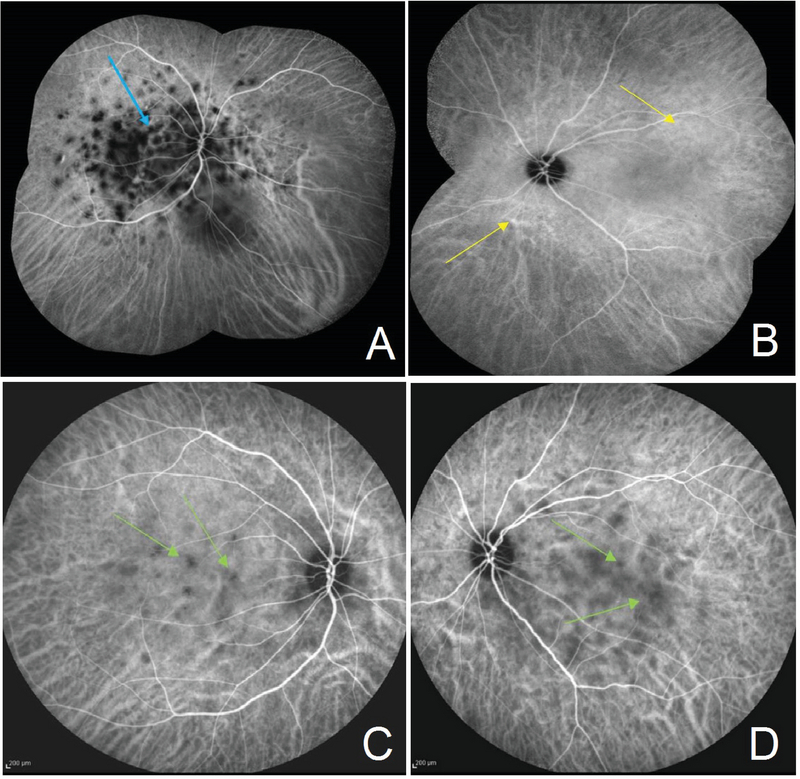
Indocyanine Green angiography at presentation. (A) Early phase of the right eye reveals multiple hypo-fluorescent dark dots in posterior pole that remains hypofluorescent until late phase (blue arrow). (B) No abnormal fluorescence in early and late phase in left eye except for mild dilation of choroidal vessels (yellow arrows). (C, D) Two months after the disease presentation during the tapering of corticosteroid: hypofluorescent dark dots in both eyes (green arrows) implying still active disease in the right eye and involvement of the left eye.

**Figure 4:**
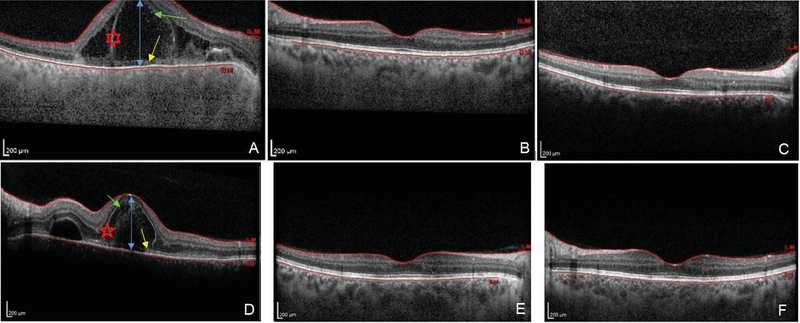
Spectral domain optical coherence tomography SD-OCT at presentation. (A) Large intraretinal cyst (red asterisk), high macular retinal detachment (blue arrow), subretinal hyperreflective membranous structure (yellow arrow), high reflective dots (green arrow). (B) Left eye: normal. (C) Complete resolution of intraretinal cyst and subretinal hyperreflective membranous structure (D) but involvement of the left eye in month 2 during the tapering of the treatment. (E, F) Normal SD-OCT of two eyes after adequate treatment.

A 44-year-old woman with progressive and painless visual blurring of the right eye for two days presented to the emergency department of the Khatam-al-Anbia Eye Hospital, affiliated to the Mashhad University of Medical Sciences, Mashhad, Iran. She had no history of ocular surgery or trauma. Her medical, ocular, and drug histories were unremarkable. The best-corrected visual acuities (BCVAs) of the right and left eyes were 20/50 (logMAR: 0.4) and 20/20 (logMAR: 0), respectively. Her slit-lamp examination result was normal. Intraocular pressure was 14 mm Hg in both eyes. Fundus examination revealed optic disc swelling and multiple serous retinal detachments in the right eye and normal left retina. Retinal imaging revealed characteristic features of VKH in the right eye. FA showed focal areas of delayed choroidal perfusion, multifocal areas of pinpoint leakage, pooling, and optic nerve staining (Figures 1 and 2). ICGA disclosed early hyper-cyanescence of the stromal choroidal vessels, which led to late hyper-cyanescence in both eyes and multiple hypofluorescent dark dots in the right eye [Figure 3 (A and B)]. The EDI-OCT findings included choroidal thickening, loculated spaces of the subretinal fluid with bands, high macular retinal detachment, subretinal membranous structure, and hyper-reflective dots in subretinal fluid [Figure 4 (A and B)]. Results of laboratory tests, including complete blood cell count (CBC), sedimentation rate, C-reactive protein, syphilis serology, toxoplasma serology, PPD (purified protein derivative) skin test, hepatitis serology, and autoimmunity markers (ANA [Antinuclear Antibody], RF [rheumatoid factor], ACE [angiotensin converting enzyme]), and chest radiography were negative.

The initial diagnosis was VKH with unilateral clinical presentation and subclinical involvement of the left eye disclosed on ICGA. The patient was treated with intravenous high-dose corticosteroid (pulse methylprednisolone 1 g/day) followed by oral prednisolone (1 mg/kg). VA and OCT findings significantly improved. Two months later and during rapid tapering of the corticosteroid dose due to systemic complications, VA of the left eye decreased, associated with anterior chamber and vitreous cellular reaction. The notable finding was the presence of vitreous and anterior chamber cells in the left eye, which was very different from the condition of the right eye in the first presentation with an absence of cellular reaction in anterior chamber and vitreous. Fundoscopic examination revealed multiple serous retinal detachments. Slit-lamp examination and fundoscopy of the right eye had normal results [Figure 3 (C and D), Figure 4 (C and D)]. Therefore, the dose of prednisolone was increased, and methotrexate (MTX) 12.5 mg/week was added. VA, clinical and paraclinical examination results significantly improved afterwards. The disease was in complete remission 12 months after treatment with MTX and tapering of the prednisolone [Figure 4 (E and F)].

##  DISCUSSION

VKH typically presents as a bilateral symmetric granulomatous uveitis. Here, we introduce a case with unilateral clinical presentation, who had abnormalities in ICGA of both eyes. This implies the importance of ICGA in the diagnosis and treatment of VKH.

VKH is diagnosed based on clinical and angiographic findings. Although according to the current revised diagnostic criteria, bilateral ocular involvement is necessary for the diagnosis of VKH, there are a few reports of unilateral or asymmetrical ocular involvement.^[9,10,12,13]^ Our patient had clinical and paraclinical features compatible with VKH except for unilaterality at initial presentation.

In this patient, except for headache, dysacusia, and mild hearing loss, review of systems and laboratory tests were normal. Ophthalmic examination, FAG, and ICGA findings were typical of VKH: serous retinal detachment, focal areas of delayed choroidal perfusion, multifocal areas of pinpoint leakage, pooling, and optic nerve staining on FAG; hypofluorescent dark dots on ICGA; and choroidal thickening, loculated spaces of subretinal fluids with bands, high macular retinal detachment, subretinal membranous structure, and hyper-reflective dots on SD-OCT. The differential diagnosis of VKH includes infectious diseases (bacteria, fungal, tuberculosis, and syphilis), malignancies (intraocular lymphoma, diffuse uveal lymphoid hyperplasia, systemic lymphoma, or leukemia), and inflammatory diseases (posterior scleritis, sarcoidosis, acute posterior multifocal placoid pigment epitheliopathy, and multiple evanescent white dot syndrome choroidopathy). These diseases can be excluded on the basis of clinical manifestations, disease course, systemic medical workup, and ophthalmic imaging findings.^[9,10,12,13]^

There was a significant improvement in BCVA and OCT findings in this case following the administration of high-dose intravenous corticosteroid. During the rapid tapering of the corticosteroid due to systemic complications, fellow eye involvement appeared clinically. Increasing the dose of corticosteroid and addition of MTX caused visual acuity and fundus features to improve markedly. High-dose corticosteroid is the mainstay treatment of VKH. Treatment must be continued with gradual tapering of the oral corticosteroid over at least six months, along with introduction of corticosteroid-sparing agents such as MTX, mycophenolate mofetil, and cyclosporine A. Poor responders to corticosteroids and other immunomodulatory agents would benefit from biological agents such as rituximab and infliximab.^[8,9,10,13]^

In summary, VKH can present with atypical features such as unilateral or asymmetrical signs and symptoms. Meticulous diagnosis is essential for prompt initiation of corticosteroids and immunomodulatory agents, as aggressive and rapid initiation of treatment is necessary to control the inflammation and thereby prevent the development of sequela and visual disability. Imaging modalities, especially ICGA, can be invaluable in the diagnosis of VKH with unilateral presentation, which shows subtle subclinical involvement.

##  Financial Support and Sponsorship

Nil.

##  Conflicts of Interest

There are no conflicts of interest.
